# A 12-week app-based multidomain intervention ameliorates cognitive performance in patients with mild cognitive impairment: preliminary results of the MEMODIO_APP@CARE RCT

**DOI:** 10.1192/j.eurpsy.2025.1445

**Published:** 2025-08-26

**Authors:** G. Nelles, T. Steinmann, D. Stein, V. Weil, A. Bicu, C. Polidori

**Affiliations:** 1 Neuromed-Campus, Cologne; 2 memodio GmbH, Potsdam; 3 Radiation Oncology, University Medical Center Mannheim, Medical Faculty Mannheim, University of Heidelberg, Mannheim; 4 Department II of Internal Medicine and Center for Molecular Medicine Cologne, University of Cologne, Faculty of Medicine and University Hospital Cologne, Cologne, Germany

## Abstract

**Introduction:**

The *Lancet Commission on dementia prevention, intervention and care* and the *WHO Guidelines on risk reduction of cognitive decline and dementia* provide evidence-based recommendations on lifestyle behaviours and interventions to delay or prevent cognitive decline and dementia. Due to demographic change and because new developments in pharmacotherapy are not suitable for all patients, there is a great need for non-pharmacological interventions. The MEMODIO app was developed to provide a multi-domain therapy for users with Mild Cognitive Impairment (MCI) and dementia. A screenshot of the app is shown in Image 1.

**Objectives:**

The aim of this study was the exploratory evaluation of whether an app-based therapy can improve cognitive function in patients with MCI and mild dementia. For the interim analysis as of October 2024, the results of the MCI study arm are reported (n=42).

**Methods:**

One hundred forty patients with confirmed diagnosis of MCI [Montreal Cognitive Assessment (MoCA) score 21-25] or Mild Dementia (MoCA score 14-20) were randomized to an intervention (IG) or standard of care (SoC) group. IG patients received SoC plus the MEMODIO app, providing cognitive and physical exercises, as well as psychoeducation on a brain-healthy diet and risk factors for cognitive decline, for 12 weeks. MoCA, Amsterdam Instrumental Activity of Daily Living Questionnaire - Short version (A-IADL-Q-SV) Dementia-Related Quality of Life (DEMQOL) as well as Physical Activity Questionnaire (PAQ 50+) were collected at baseline and study end.

**Results:**

In the MCI group, the mean age was 71 years, 20 out of 42 patients were female. Preliminary outcomes are shown in Images 2 and 3.

The preliminary analysis on MCI patients (mean age: 71 y ±9,49 SD, 48% were female) showed statistically significant improvements in MoCA in the IG (-0.84±3.5 SoC vs. 1.96±2.7 IG, p=0.006). Quality of life, physical activity and activities of daily living at the time point of the interim evaluation were not significantly different from baseline, but the activity level measured by PAQ showed a non-significant improvement in the SoC group.

**Image:**

**Figure:**
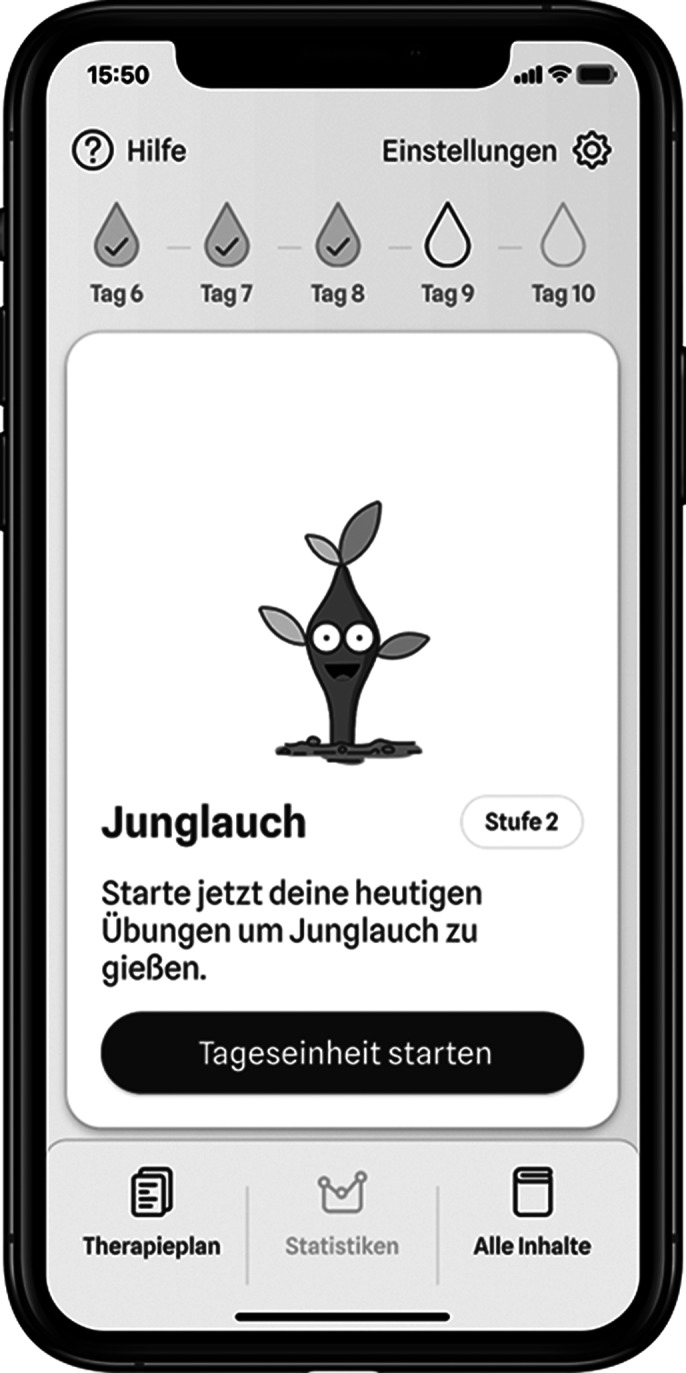

**Image 2:**

**Figure:**
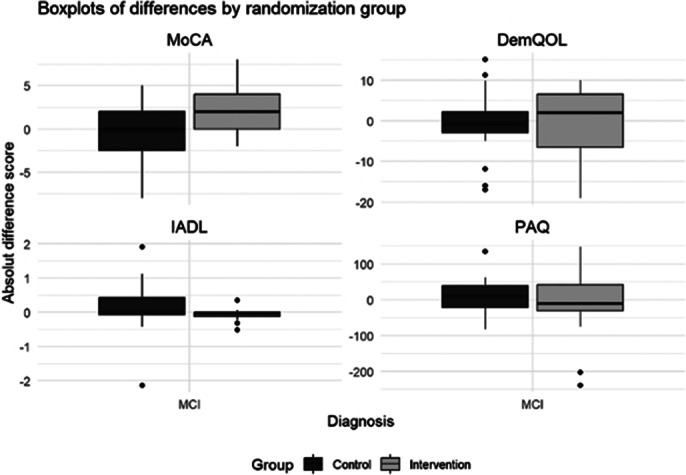

**Image 3:**

**Figure:**
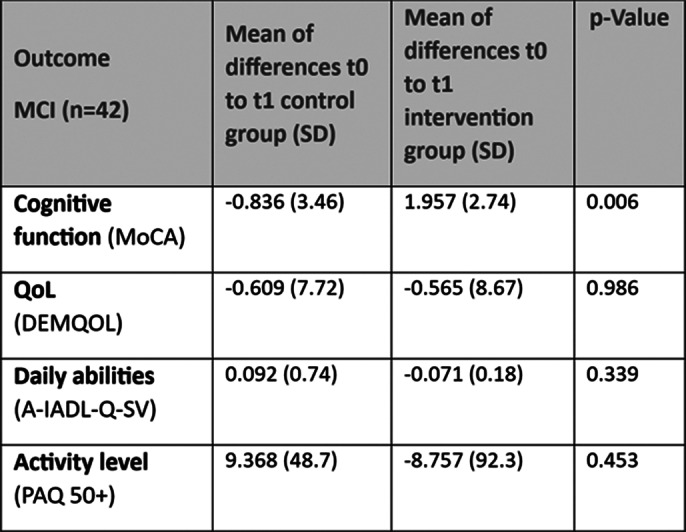

**Conclusions:**

In this RCT, a significant improvement in cognition was shown in MCI-patients using the MEMODIO app compared to those receiving SoC alone. Further analyses are ongoing.

**Disclosure of Interest:**

G. Nelles Grant / Research support from: PI of MEMODIO@APP_CARE, T. Steinmann Employee of: memodio GmbH, D. Stein Shareolder of: memodio GmbH, V. Weil Employee of: memodio GmbH, A. Bicu: None Declared, C. Polidori: None Declared

